# The Role of Extracellular Vesicles in Idiopathic Pulmonary Fibrosis Progression: An Approach on Their Therapeutics Potential

**DOI:** 10.3390/cells11040630

**Published:** 2022-02-11

**Authors:** Alma Aurora Ramírez-Hernández, Juan Manuel Velázquez-Enríquez, Jovito Cesar Santos-Álvarez, Armando López-Martínez, Edilburga Reyes-Jiménez, Gabriela Carrasco-Torres, Karina González-García, Verónica Rocío Vásquez-Garzón, Rafael Baltierrez-Hoyos

**Affiliations:** 1 Laboratorio de Fibrosis y Cáncer, Facultad de Medicina y Cirugía, Universidad Autónoma Benito Juárez de Oaxaca, Oaxaca de Juárez 68120, Mexico; aramih_09@cecad-uabjo.mx (A.A.R.-H.); juanmanuelvela_enriquez@cecad-uabjo.mx (J.M.V.-E.); jovitocesarsa@cecad-uabjo.mx (J.C.S.-Á.); armandoloopez37@cecad-uabjo.mx (A.L.-M.); edilreyesjimnez@cecad-uabjo.mx (E.R.-J.); k.igg@cecad-uabjo.mx (K.G.-G.); 2 Departamento de Nanociencias y Nanotecnología, Centro de Investigación y de Estudios Avanzados del IPN, Av. IPN 2508, la laguna Ticomán, Ciudad de Mexico 07360, Mexico; gabriela.carrasco@cinvestav.mx; 3 CONACYT-Facultad de Medicina y Cirugía, Universidad Autónoma Benito Juárez de Oaxaca, Oaxaca de Juárez 68120, Mexico; vrvasquezga@conacyt.mx

**Keywords:** extracellular vesicles, fibroblasts, macrophages, alveolar epithelial cells, mesenchymal stem cells, therapeutic

## Abstract

Idiopathic pulmonary fibrosis (IPF) is a fibrosing interstitial lung disease of unknown etiology. Different types of cells are involved in fibrogenesis, which is persistently physical and molecular stimulation, either directly or by interacting with bioactive molecules and extracellular vesicles (EVs). Current evidence suggests that EVs play an essential role in IPF development. EVs are released by a variety of cells, including fibroblasts, epithelial cells, and alveolar macrophages. In addition, EVs can transport bioactive molecules, such as lipids, proteins, and nucleic acids, which play a pivotal role in cellular communication. Several proposed mechanisms show that an acceptor cell can capture, absorb, or interact with EVs through direct fusion with the plasma membrane, ligand–receptor interaction, and endocytotic process, modifying the target cell. During fibrogenesis, the release of EVs is deregulated, increases the EVs amount, and the cargo content is modified. This alteration is closely associated with the maintenance of the fibrotic microenvironment. This review summarizes the current data on the participation of EVs secreted by the cells playing a critical role in IPF pathogenesis.

## 1. Introduction

### 1.1. Idiopathic Pulmonary Fibrosis

Idiopathic pulmonary fibrosis (IPF) is fibrosing interstitial pneumonia of unknown etiology. It is a chronic and progressive disease characterized by aberrant deposition of extracellular matrix (ECM) in the lung parenchyma that deforms the alveolar architecture, causing respiratory failure as it decreases lung function and gas exchange, which can eventually cause death [1]. The synergistic effect of both internal and external factors are required for the initiation and progression of IPF mainly, in subjects with a genetic predisposition. Tissue alterations are established through recurrent lesions in the pulmonary epithelium superimposed on the accelerated aging of the epithelium itself, leading to an aberrant repair of lung parenchyma and causing excessive accumulation of ECM and all clinical features of the disease [1,2].

### 1.2. Epidemiology

To date, epidemiological data of IPF are unknown due to both inconsistencies in the estimation criteria and different methodologies used. However, a systematic review has summarized the data published between 1990 and 2020 and estimated a global prevalence of 3.3 to 45.1 per 100,000 inhabitants, although the specific prevalence per country tends to be different depending on the geographic region. For example, the prevalence in North America is estimated between 24.0 and 29.8, in Europe, between 3.3 and 25.1, while in Asia-Pacific countries, it is between 5.7 and 45.1, per 100,000 inhabitants [3]. Thus, the global incidence is estimated in a range of 6.8 to 16.3 per 100,000 inhabitants, and an estimated rate of 2.8 to 9.3 per 100,000 per year in North America and Europe. Asia and South America have significantly lower rates [4]. IPF is a disease of poor prognosis with an average survival of 3–5 years after diagnosis; it occurs mainly in people over 65 years old and occasionally in young people. Interestingly, IPF is three times more common in men than in women, and it has been established that between 0.5 and 3.7% of IPF cases are familial [5].

### 1.3. Clinical Manifestations

The initial clinical symptoms of IPF are not well-known, but consist of progressive exertional dyspnea that may or may not is accompanied by a dry cough, that in many cases, is initially attributed to either aging, deconditioning, or other comorbidities, such as a history of smoking or obesity, which delays a timely diagnosis [6]. At the early disease stage, “velcro-like” crepitations can occur, but cyanosis and marked respiratory distress can be observed with the disease progression. In addition, in 25–50% of cases, finger clubbing and even clinical features suggesting an underlying connective tissue disease can also be observed [7]. Some of the main morbidities associated with IPF are emphysema, pulmonary hypertension, cardiovascular diseases, gastroesophageal reflux diseases, and lung cancer, which contribute to the clinical manifestations being even more heterogeneous [6].

### 1.4. Diagnostic

The diagnosis of IPF lacks a universal algorithm and is therefore carried out based on the criteria of the treating physician. However, for an accurate diagnosis, it requires a multidisciplinary professional team of experts who collaborate, such as pulmonologists, pathologists, and radiologists, and who use clinical practice guidelines issued by various institutions [8].

In 2018, the American Thoracic Society (ATS), the European Respiratory Society (ERS), the Japanese Respiratory Society (JRS), and the Latin American Thoracic Society (ALAT) published the latest version of the clinical practice guidelines for the diagnosis of IPF, which propose four diagnostic categories including a usual interstitial pneumonia pattern (UIP), a probable pattern of UIP, indeterminate UIP pattern, and alternative diagnosis based on high-resolution computed tomography (HRCT) of the chest, and the histopathology of surgical lung biopsy (SLB). Thus, the definitive diagnosis might consist essentially of the following criteria:

Detailed clinical history excludes other UIP causes, e.g., domestic and occupational environmental exposures, connective tissue disease (CTD), and drug toxicity.

For patients with SLB that are not indicated or available, the diagnosis is based on UIP pattern identification on HRCT, which consists of a honeycomb morphology, bronchiectasis, and ground glass opacification in the lungs.

For patients with SLB, the diagnosis is made by using specific combinations of HRCT and histopathological patterns of the biopsies, consisting of dense fibrosis patches with irregular alteration of the lung architecture presenting in honeycomb morphology [9].

### 1.5. Treatment

Currently, there is no specific and effective treatment for patients bearing IPF, but drug therapy focuses on improving symptoms, preserving lung function, minimizing the adverse effects of treatment, and increasing survival [10]. The most widely used therapeutic strategy consists of the administration of a combination of *N*-acetylcysteine (NAC) (mucolytic), prednisone (corticosteroid), and azathioprine (an immunosuppressant); however, there remains some uncertainty about the efficacy of the third one and many more therapies; moreover, it is established that they can be harmful to patients [11]. In addition, due to the progressive character of IPF, the administration of supplemental oxygen, vasodilators, and opioids is also required to counteract the most frequent and debilitating complications for the patient, such as dyspnea and cough [10].

In 2014, the United States Food and Drug Administration (FDA) approved two antifibrotic drugs, pirfenidone and nintedanib, for IPF treatment; the following year, the ATS/ERS/JRS/ALAT suggested the routine use of these antifibrotics. However, although they have been shown to increase survival and decrease mortality, they seem not to be sufficient to reverse the disease; moreover, they present several adverse effects and are very costly [12]. Therefore, lung transplantation is the last option to prolong survival and improve quality of life; however, only 66% of recipients survive longer than 3 years after transplantation due to further complications [2,13].

### 1.6. Fibrogenesis and Cellular Communication

Fibrosis is a physiological and dynamic process involved in wound healing that restores and maintains organ integrity after injury [14]. Overlapping phases generally carry out physiological fibrosis: (I) initiation, caused by an organ/tissue injury; (II) inflammation and activation of effector cells, (III) increased ECM synthesis, (IV) ECM deposition and (V) resolution, which allows remodeling the organ/tissue. However, in IPF, the existence of a stimulus of persistent damage leads to pathological fibrosis without resolution, which causes aberrant accumulation of ECM, destroys the tissue architecture, and decreases lung functionality [15,16]. It has been proposed that environmental exposures viral and bacterial infections represent the primary stimulus of persistent damage in aging dysfunctional lung epithelium; nevertheless, genetic and epigenetic factors that predispose the initiation of the fibrotic process are also needed [1,2].

In this line, once tissue damage is achieved, the release of chemoattractant molecules by the damaged epithelial cells is promoted, facilitating the recruitment of immune system cells, mainly neutrophils, macrophages, and lymphocytes. These cells open the door to the release of various inflammatory and profibrotic molecules such as transforming growth factor-beta (TGF-β), which is considered the master regulator of the fibrotic process, platelet-derived growth factor (PDGF) connective tissue growth factor (CTGF), as well as various interleukins, such as interleukin-1β (IL-1β), interleukin 6 (IL-6), interleukin-13 (IL-13), and interleukin-33 (IL-33), chemokines and non-peptide molecules, such as bioactive lipids and reactive oxygen species (ROS), which in turn recruit more immune system cells and activate effector cells creating an inflammatory environment favorable for the development of IPF [17,18]. In this sense, it has been described that inflammatory events play an essential role in developing the pathological state of IPF [18]. This inflammatory condition is divided into an early and late stage, where the early stage of the inflammatory state is partially reduced during the development of IPF to give way to the onset of the scarring process that culminates in the development of the fibrotic process in the lung tissue [18]. On the other hand, it is described that during the late stage, which is favored, the recruitment of inflammatory cells that contribute to control the excessive cell proliferation and eliminate the cellular residues supported by the phagocytosis process will result in the normal wound healing process. However, if the damage is chronic and persistent, it will result in an aberrant wound healing process. Because of the above, it has been hypothesized that late-stage inflammation may develop an essential antifibrotic role in resolving wound healing responses [19]. It has been suggested that the possible mechanisms by which this antifibrotic effect occurs are by the elimination of fibroblasts affected by the process of phagocytosis mediated mainly by macrophages recruited to the site of injury and by the secretion of interleukin-10 (IL-10) by regulatory T cells, which significantly attenuates TGF-β production and secretion [20].

It is well known that fibroblasts are the critical effector cells in fibrogenesis, which respond to mechanical and chemical stimuli that differentiate them into [21]. As a result, they acquire a secretory ECM phenotype, resistance to apoptosis, express surface receptors, and create a positive feedback loop that amplifies the differentiation of these cells by releasing various inflammatory and profibrotic molecules, mainly TGF-β; in addition, fibrocytes, pericytes, epithelial, endothelial, and mesothelial cells can also transform into myofibroblasts. Overall, different cell types orchestrate the lung microenvironment to promote crosstalk between profibrotic and antifibrotic mediators, contributing to chronic ECM deposition [18,22].

The flux of this microenvironment is essential to coordinate cellular and molecular events. Thus, its disturbance can lead to the initiation and progression of pathogenic events during IPF [23]. The lung cells are in a constant exchange of physical and molecular stimuli, either directly or through their secretion, which maintains a profibrotic microenvironment [5,24], exchanging ions and low molecular weight molecules through gap junctions and ligand–receptor interactions [25]. In addition, they release bioactive molecules such as nucleic acids, lipids, soluble proteins, and extracellular vesicles (EVs) in an autocrine, paracrine, and endocrine manner [23,24].

## 2. Extracellular Vesicles

EVs are cell fragments with a spherical morphology consisting of a lipid bilayer membrane and hydrophilic proteins, classified as exosomes and microvesicles [26,27]. Initially, they were considered waste particles, but further studies have demonstrated their usefulness and function. In 1987, the term exosome was used for the first time to describe small membrane vesicles formed by invagination of intracellular endosomes and released by exocytosis [28]. The most common procedures for their isolation and concentration have been ultracentrifugation, ultrafiltration, density gradient, and chromatography [29].

Proteins within EVs are those involved in their biogenesis, such as the endosomal classification complex necessary for transport (ESCRT) machinery. However, they also contain cytoskeletal components, transmembranal, adhesion, antigen presentation, and others proteins [30]. Additionally, they contain lipids including sphingomyelin, phosphatidylserine, cholesterol, and ceramides, among others, as well as nucleic acids such as DNA, mRNA, microRNA, circular RNA, and long non-coding RNA [29].

### 2.1. Biogenesis and Classification

EVs are classified according to their origin; thus, two groups are well-known: exosomes and microvesicles. Exosomes size is 30–100 nm in diameter, and they are produced by the membrane invagination in multivesicular bodies (MVB), which form intraluminal vesicles and can fuse with the cell membrane to release the exosomes into extracellular space. Microvesicles size is 100–1000 nm in diameter, and they are direct shoots from the plasma membrane, which are released by the action of the actin–myosin complex towards extracellular space [31].

A key proteins group involved in MVB formation is that of the ESCRT machinery, which is divided into four different groups [32]. These proteins are destined for exocytosis and transported by microtubules, and their release depends on the cell type and involves proteins such as RABs GTPases [33]. After transport and support to the cell membrane, the MVB proteins are coupled to soluble N-ethylmaleimide sensitive factor attachment protein receptor (SNARE) proteins, where small GTPases play a relevant role. Finally, when fusion takes place and exosomes are released, they interact with either ECM proteins or the receptors in other adjacent cells, modifying the response to stimuli from the microenvironment [34].

### 2.2. Molecular Mechanisms of EVs Release

**Figure 1:**
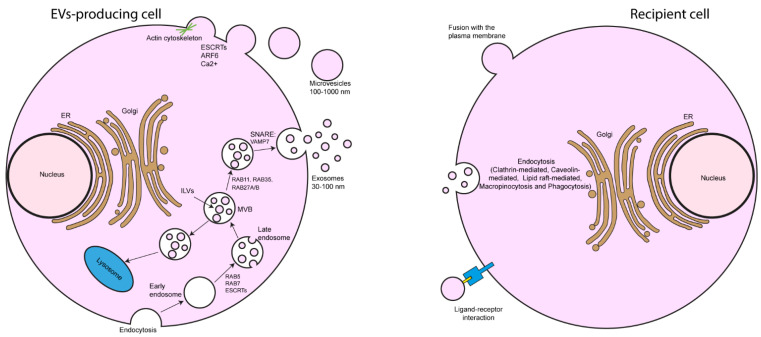
Biogenesis-uptake of EVs. Exosomes are generated through the endocytic pathway by forming an early endosome that matures to produce a late endosome or MVB. Upon budding at its endosomal membrane, it can originate small vesicles called intraluminal vesicles. This can be driven for degradation in the lysosome or interact with the cell plasma membrane to fuse, allowing the release of exosomes (30–100 nm) into the extracellular medium. Microvesicles (100–1000 nm) are released by direct gemination from the cell plasma membrane. Once EVs are released into the extracellular medium, they can interact with a recipient cell through various mechanisms, including direct fusion with the plasma membrane, ligand–receptor interaction, and endocytosis processes including clathrin, caveolin, and lipid raft-mediated endocytosis, micropinocytosis, and phagocytosis.

Proteins involved in the mechanisms of exosomes release include Rab GTPases such as RAB11, RAB35, RAB27 A, and B. RAB11 and RAB35 facilitate the fusion of the MVB with the plasma membrane [35]. RAB27A and B function in coupling multivesicular endosomes in the plasma membrane [33]. Another protein is vesicle-associated membrane protein 7 (VAMP7), a protein from the SNARE group, which stimulates exosomes containing acetylcholinesterase [36]. It has been hypothesized that the release of exosomes differs from that of microvesicles because of the external budding and external fission of the plasma membrane, which can be caused by a rearrangement of phospholipids and components contraction of the cytoskeleton, including actin and myosin [37]. Nabhan and coworkers reported that the recruitment of ESCRT-I subunit and tumor susceptibility gene 101 (TSG101) protein into the plasma membrane is through arrestin domain-containing protein 1-mediated microvesicles (ARMMs), which result in their release [38]. Other external factors that may trigger the microvesicles release are the entry of calcium, which modifies the distribution of membrane phospholipids, and hypoxia, which works through a mechanism dependent on the inducible hypoxia factor [39,40] (Figure 1).

EVs are released by various cells types and can transport bioactive molecules such as lipids, proteins, and nucleic acids, and they play an essential role in cellular communication processes. In target cells, these molecules act as signaling complexes promoting diverse cellular processes in both physiological and pathological conditions. However, it is believed that their functioning is extensive, but they have not been fully described yet [41,42]. Some of the processes where EVs have been involved are the immune system, carcinogenesis, cardiovascular, and chronic lung diseases [41,42,43,44].

### 2.3. Mechanisms of EVs Binding to the Target Cell Membrane

Several mechanisms have been proposed where an acceptor cell can capture, absorb or interact with EVs through direct fusion with the plasma membrane, ligand–receptor interaction, and endocytotic process (Figure 1) [45,46]. Nevertheless, the mechanism for capturing EVs depends on the cell type, composition of the membrane proteins, and physiological state [45].

## 3. The EVs Release Is Deregulated in IPF Development

An important difference between the molecular load and amount of EVs has been observed in the microenvironment of the diseased lung, which has been attributed to the IPF development and different stress conditions such as inflammation and oxidative stress [42,47]. For instance, it has been demonstrated that stimulation with cigarette smoke extract (CSE) and acrolein promotes an increase of EVs secretion in human lung epithelial BEAS-2B cells; interestingly, this effect was reduced by the antioxidant agents NAC and reduced glutathione (GSH) [47]. Furthermore, proteomic analysis of these EVs showed that 26.3% and 8.0% of proteins were significantly up-regulated and down-regulated, respectively, in CSE-treated cells compared to control. Interestingly, results showed that proteins involved in hemostasis and platelet activation, signaling, and aggregation were up-regulated, demonstrating that EVs released from CSE-treated cells maintain procoagulant properties [48]. In addition, it was also shown that this secretion is increased in both experimental and human IPF. Authors found a higher EVs number, as well as a significant increase in protein concentration of EVs isolated from bronchoalveolar lavage fluid (BALF) of mice undergoing bleomycin-induced pulmonary fibrosis compared to healthy mice; these results were replicable in human IPF, where a significant increase in the number of EVs was observed in the BALF of IPF patients compared to healthy controls [42].

Besides, several reports have shown that lung fibroblasts express an IPF-associated senescent phenotype release significantly more EVs than non-senescent lung fibroblasts. Likewise, it was shown that the stimulation of non-senescent lung fibroblasts with TGF-β1 induces an increase in EVs production. Similarly, lung fibroblasts from IPF were found to generate a more significant number of EVs than those from healthy subjects [49,50].

Moreover, we have characterized a differential pattern of protein expression contained within EVs secreted by fibroblasts isolated from IPF lungs and observed that some proteins involved in the promotion of various fibrogenic processes, such as tenascin-c (TNC), insulin-like growth factor-binding protein 7 (IGFBP7), fibrillin-1 (FBN1), collagen alpha-2 (I) chain (COL1A2), collagen alpha-1 (I) chain (COL1A1), and lysyl oxidase homolog 1 (LOXL1), were significantly up-regulated in EVs cargo, indicating that proteins carried out by EVs could play a key role during fibrogenesis associated with the IPF pathogenesis [51].

## 4. EVs and Their Involvement in Cellular and Molecular Mechanisms Associated with IPF

**Figure 2:**
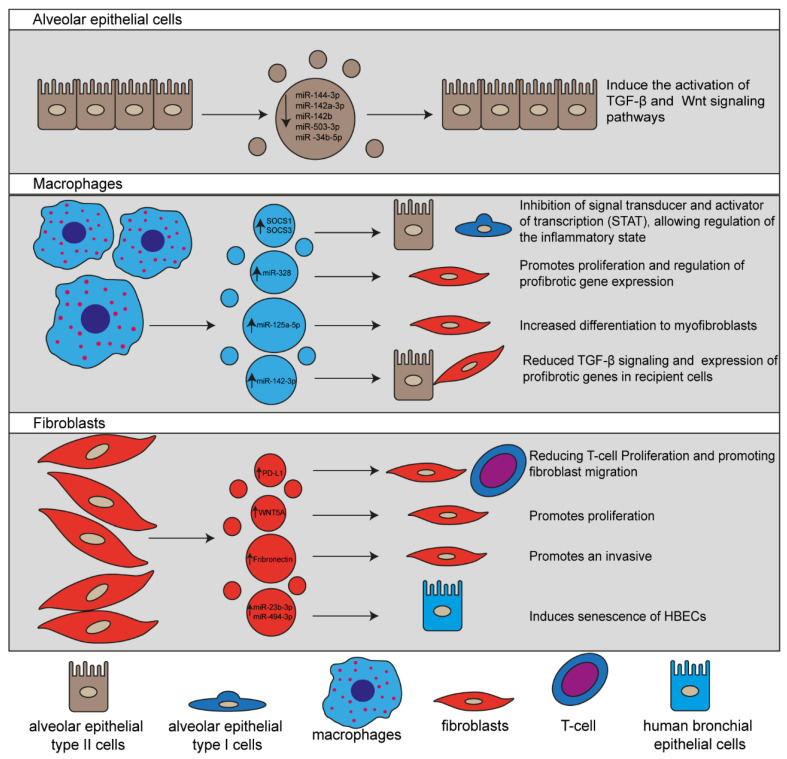
Schematic representation of EVs released by different lung cells involved in IPF pathogenesis. EVs and their cargo molecules regulate the main signaling pathways associated with profibrotic processes facilitating the IPF progression.

Several reports have proposed that EVs function as messengers and critical players in the pathogenesis of IPF [42]. However, the mechanism promoting lung fibrogenesis remains to be fully described. Therefore, further researches are still needed to elucidate both cellular and molecular mechanisms involved in this process. Below, we briefly summarize the current evidence on the involvement of EVs secreted by cells participating in the development of IPF (Figure 2).

### 4.1. Cells Involved in IPF Development

The involvement of cells in the fibrogenesis of IPF has been extensively studied; nevertheless, the exact mechanism of EVs in promoting cellular communication leading to IPF development and evolution remains unclear [52,53]. Current knowledge on the pathogenesis of IPF has demonstrated that during the early stage of the disease, chronic tissue injury promote apoptosis of type I alveolar epithelial cell (AEC-I), recruitment of inflammatory cells, mainly macrophages and neutrophils, activation of type II alveolar epithelial cell (AEC-II) that regenerate damaged cells, and the release of proinflammatory cytokines that promote the recruitment, activation, and proliferation of fibroblasts [5,52]. Thus, fibroblasts are the most important cells in IPF development since they undergo differentiation into myofibroblasts facilitating their capability to produce and secrete ECM components [52].

#### 4.1.1. EVs Released by Alveolar Epithelial Cells

A critical step during IPF development is the persistent injury of AEC-I and II by environmental factors. During this process, AECs may undergo apoptosis, release inflammatory mediators. EVs that strongly contribute to IPF development [52,54]. Recently, it has been reported that syndecan-1 protein is increased in both lung epithelium of IPF patients and murine bleomycin-induced IPF models. Likewise, authors also demonstrated that syndecan-1 plays an essential role in the packaging of antifibrotic miRNAs in EVs, including miR-144-3p, miR-142a-3p, miR-142b, miR-503-3p, and miR-34b-5p, which have shown diverse effects on signaling pathways associated with fibroproliferation and fibrogenesis, suggesting that those miRNAs play a pivotal role in the regulation of IPF progress. Moreover, they observed that by incubating AEC-II with EVs isolated from wild-type and Sdc1^−/−^ mice models of IPF induced with bleomycin, EVs from wild-type mice act as a profibrotic signal promoting the activation of different signaling pathways involved in fibrotic processes, such as TGF-β and Wnt as compared with the effect of EVs isolated from Sdc1^−/−^ mice [54].

#### 4.1.2. EVs Secreted by Macrophages

Alveolar macrophages secrete EVs to transport the suppressor of cytokine signaling 1 and 3 (SOCS1 and SOCS3, respectively). Consequently, they are primarily taken up by AECs, resulting in the inhibition of signal transducer and activator of transcription (STAT), which regulates the inflammatory response, indicating that EVs derived from macrophages are involved in the pulmonary homeostasis [55].

On the other hand, Yao et al. have demonstrated that macrophage-derived EVs bearing an M2 phenotype transport miR-328 and that this miRNA charge participates in promoting IPF progression by negatively regulating family with sequence similarity 13, member A (FAM13A) and as a result, inducing lung fibroblasts proliferation and overexpression of collagens 1A, 3A, and alpha-smooth muscle actin (α-SMA) [56]. Recently, it has been demonstrated that EVs secretion increases in the supernatant of silica (SiO_2_)-stimulated macrophage. Moreover, lung fibroblasts incubated with these EVs are differentiated into myofibroblasts by inducing endoplasmic reticulum stress, which results in a significant increase in proliferation, migration, and expression of fibrotic proteins, such as collagen I and α-SMA [57]. Besides, recently, it was reported that miR-125a-5p is upregulated in EVs of silica (SiO_2_)-stimulated macrophage, and when lung fibroblasts are incubated with these EVs, α-SMA is overexpressed, and TGF-β is activated to mediate their differentiation into myofibroblasts through downregulation of Smurf1 [58].

Contrarily, it has been described that miR-142-3p contained in EVs derived from macrophages are responsible for suppressing the profibrotic activation of both AECs and lung fibroblasts mediated by TGF-β as well as of repressing the expression of profibrotic genes, such as *COL1A1*, *COL1A2*, and *TGF-β* [59].

#### 4.1.3. EVs Released by Fibroblasts

Fibroblasts are in charge of restoring the ECM components after injury; nonetheless, in the pathogenesis of IPF, this capability is altered by the persistent exposition to profibrotic factors, which leads to increased production of ECM components [52]. Circulating proteins, such as TGF-β and PDGF, have been described as the main profibrotic factors in the fibroblasts activation process, and the role of EVs as carriers of signals promoting fibroblasts activation has also been studied [40,52]. Recently, Martin-Medina et al. demonstrated that Wnt family member 5A (WNT5A) protein is overexpressed in EVs isolated from both BALF of IPF patients and an IPF bleomycin-induced murine model. Furthermore, they reported that TGF-β-mediated activation of primary human pulmonary lung fibroblasts (PHLF) is closely associated with an increased level of WNT5A and its release within EVs. They also demonstrated that EVs isolated from BALF of IPF patients and cell culture supernatant of PHLF activated with TGF-β increased lung fibroblasts proliferation in a WNT5A-dependent manner [42].

Alternatively, fibroblasts from IPF patients have been reported to secrete EVs fibronectin-enriched, which promote an invasive phenotype in recipient fibroblasts through the interaction of fibronectin with α5β1 integrin, triggering the activation of signaling pathways associated with cell invasion processes, such as Src family kinases and focal adhesion kinase [49].

A recent study has demonstrated that after stimulation of human and murine lung fibroblasts with TGF-β, the programmed death-ligand 1 (PD-L1) is overexpressed in EVs derived from fibroblasts; PD-L1 acts as a paracrine mediator of the immunosuppression by reducing T-cell proliferation while promoting fibroblasts migration [60]. Another investigation group has demonstrated that EVs secreted by lung fibroblasts isolated from IPF patients promote mitochondrial damage and senescence of human bronchial epithelial cells (HBECs). In addition, they evidenced that these EVs contain a high amount of miRNAs, including miR-23b-3p and miR-494-3p, that suppress sirtuin 3 (SIRT3) expression, thus favoring epithelial phenotypic changes [50].

## 5. Involvement of MSCs in Lung Repair

Previous studies have proposed that mesenchymal stem cells (MSCs) are responsible for maintaining vascular homeostasis and facilitating repair; contrarily, studies performed in lung tissue have reported that MSCs participates in the restoration of injured endothelium through the release of paracrine mediators, such as EVs and activation of different signaling pathways, including (IL-6), interleukin 6 receptor subunit alpha (IL-6RA), Janus kinase (JAK), signal transducer and activator of transcription 3 (STAT3) [61,62]. In vitro studies demonstrated that human lung resident MSCs (Lr-MSCs) can decrease fibroblasts proliferation and increase the ability to induce wound closure in alveolar epithelial cells A549 [63], and decrease collagen deposition, T-cell, granulocyte and B-cell infiltration into the alveolar space, and limit T-cell proliferation in a bleomycin-induced model of pulmonary fibrosis [64]. Another study showed that menstrual blood-derived stem cells (MenSCs) inhibit the inflammatory response after induction of lung damage with lipopolysaccharide (LPS) [65]. In this respect, recent studies have been focused on evaluating the therapeutic potential of MSCs, as well as EVs and molecules secreted by these cells during IPF [66,67].

### 5.1. EVs Released by MSCs as Therapeutic Mediators

**Figure 3:**
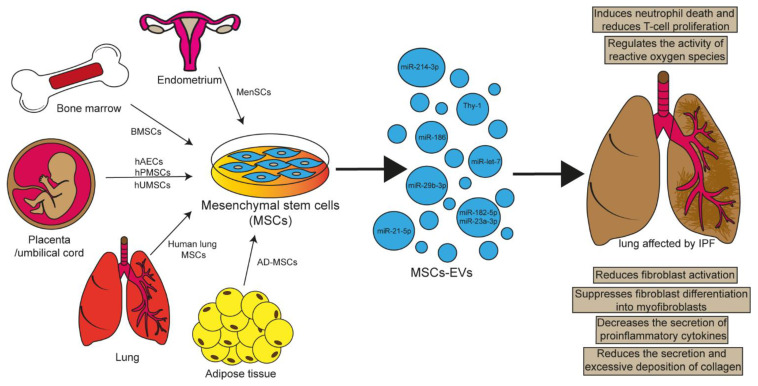
Function of stem cell-derived EVs. MSCs from either menstrual blood, bone marrow, placenta, or adult organs, such as the lung, exert a therapeutic effect through their secretome, which contains EVs that transport miRNAs that regulate several processes, including inflammation, by decreasing neutrophil recruitment, reducing cell proliferation, decreasing the apoptosis of injured epithelium and proinflammatory cytokines secretion, and reducing fibroblasts activation by transferring miRNAs.

Since stem cells protect the lung from damage, several types of research have been focused on studying the role therapeutic of the mesenchymal stem cell-derived EVs (MSC-EVs) of various tissues, including the umbilical cord, menstrual blood, bone marrow, placenta, or adult organs, such as the lung (Figure 3). This is because MSC-EVs have been reported to mimic the therapeutic capabilities of MSCs and maintain a reduced immunogenic property [68,69]. In this respect, it has been described that MSC-EVs might regulate proliferation, maturation, polarization, and cell migration, as well as modulate inflammatory cytokines, transcription factors, and miRNAs function in the cell microenvironment. Although the repairing mechanism has not been fully elucidated, evidence indicates that the reprogramming mechanism of lung cells induced by damage might be associated with miRNA-delivery, such as miR-186, which is contained in human bone marrow mesenchymal stem cell-derived EVs (BMSC-EV) and might reduce fibroblasts activation by downregulating SRY-box transcription factor 4 (SOX4) and Dickkopf 1 (DKK1), an inhibitor of the WNT signaling pathway, resulting in collagen reduction [68,70,71]. However, the studies and results obtained on the therapeutic potential of MSC-EVs in IPF have been studied mainly in murine and in vitro models (Table 1 and Table 2). Therefore, there are still many limitations to the possible therapeutic application of MSC-EVs in human IPF.

#### 5.1.1. hUMSC-EVs

MSC-EVs can reduce the recruitment of inflammatory cells, cytokine levels, and the protein concentration in alveoli, which introduce pulmonary edema and decrease intrinsic and extrinsic apoptosis pathways in the injured epithelium [71]. In addition, studies have suggested that MSC-EVs obtained from different tissues facilitate the resolution of inflammatory response and apoptosis in the lung; some models have been used to demonstrate the ability of MSC-EVs: the chronic asthma model where hUCMSC-EVS obtained under hypoxic conditions, they observed that had a greater capacity to suppress airway inflammation in asthmatic mice, as well as the decrease of profibrogenic markers such as α-SMA, collagen-1, and TGF-β-p-smad2/3 signaling pathway; in the chronic obstructive pulmonary disease model, they also found that hUMSC-EVS decreased inflammation, reduced damage to the alveolar septum, and reduced the expression levels of the proinflammatory transcription factor nuclear factor kappa B (NF-κB) [73,84,85].

#### 5.1.2. hAEC-EVs

EVs derived from human amnion epithelial cells (hAECs) modulate the antifibrotic, immunomodulatory, and regenerative properties implicated in IPF initial stage; thus, regulating the response of immune cells by inducing apoptosis of neutrophils and by inhibiting the proliferation of T cells through a mechanism dependent of the transfer of anti-fibrotic proteins and miRNAs contained in EVs, despite the effect of these EVs, the molecular mechanisms remain poorly elucidated, so further studies are needed to understand which signaling pathway hAEC-EVs induce α-SMA reduction, increase the percentage of AEC-II and inhibit myofibroblasts differentiation and their immunomodulatory activity [79].

#### 5.1.3. AD-MSCs-EVs

Various MSC-EV therapies have been investigated as potential therapeutic strategies in experimental models of lung diseases; among them, the therapeutic potential of adipose-derived mesenchymal stem cell-derived EVs (AD-MSC-EVs) has been evaluated. Recent studies demonstrated that these AD-MSC-EVs positively attenuate acute lung injury by transferring miR-27a-3p into alveolar macrophages and promoting macrophage polarization towards an M2 phenotype [78]. Moreover, MSC-EVs function might depend on cell age, although recent studies have shown that either young or aged cells have similar physical and phenotypic properties. Nevertheless, it has been demonstrated that in LPS-induced lung damage, the EVs secreted by young cells alleviate tissue injury and have greater capability to suppress the activation of proinflammatory genes, such as IL-6, IL-1β, and tumor necrosis factor alpha (TNF-α), while EVs of aged cells do not promote a protective response and do not alter macrophage phenotypes [77].

#### 5.1.4. BMSC-EVs

Bleomycin-induced pulmonary fibrosis treated with intravenous BMSC-EVs showed a reversal of bleomycin-induced damage by decreasing collagen deposition [86]. The mechanisms remain to be elucidated; however, other studies have shown that intravenous administration of BMSC-EVs inhibits vascular remodeling and pulmonary hypertension by suppressing hypoxic STAT3 signaling [74].

Wan X et al. have reported that miR-29b-3p is overexpressed in BMSC-EVs and inhibits proliferation, migration, differentiation, and invasion of the LL29 cell line (lung fibroblasts bearing IPF cell line) by suppressing frizzled-6 (FZD6) expression and generating antifibrotic effects in vitro and in vivo [75]. Furthermore, BMSCs-EVs suppress TGF-β-induced normal and IPF fibroblasts differentiation into myofibroblasts by decreasing α-SMA, fibronectin, and collagen III expressions. Moreover, the BMSC-EVs fibroblasts uptake is due to the presence of Thy-1, a cell surface-expressed in MSC-EVs; Thy-1 interacts with the integrins β1, β3, and β5, promoting the EVs capture [81].

Some research has shown that hBMSC-EVs modulate the activity of cells involved in immune response, such as dendritic cells. The treatment of these cells with hBMSC-EVs has shown an increased production of anti-inflammatory cytokines, mainly TGF-β, and reduced synthesis of proinflammatory cytokines, such as IL-6 and IL-12, after LPS stimulation [82]. MSC-EVs inactivate NF-κB and hedgehog pathways by transferring specific miRNAs, such as miR-182-5p and miR-23a-3p, and targeting inhibitor of nuclear factor kappa B kinase subunit Beta (Ikbkb) and ubiquitin carboxyl-terminal hydrolase 5 (Usp5), which reverts EMT in LPS-treated MLE-12 cells (murine lung epithelial cell line;) [76]. Zulueta et al. have demonstrated that the effects of MSC-EVs on IB3-1 cells (Adeno-associated virus-transformed human bronchial cell line) could involve peroxisome proliferator-activated receptor gamma (PPAR-γ) activation. This transcription factor interacts with NF-κB and heme oxygenase 1 (HO-1), regulating the inflammatory cascade through the nuclear translocation of NF-κB [83].

#### 5.1.5. MenSC-EVs

Due to the effect that MenSCs have shown as therapeutic mediators in several diseases, Sun and coworkers evaluated the potential of EVs derived from menstrual blood stem cells (MenSC-EVs), and have shown a protective effect in a bleomycin-induced IPF model by reducing collagen deposition and regulating ROS activity on alveolar epithelial cells through the transport of miR-Let-7; this is possibly through inhibiting ROS/mtDNA and fibrosis signaling cascades [80].

## 6. Conclusions and Perspectives

In conclusion, IPF is a chronic progressive pathology of unknown etiology, poor prognosis, and high mortality rate. This disease involves a heterogeneous group of cells such as AEC-I, AEC-II, macrophages, and fibroblasts that orchestrate a pathological state mediated by a microenvironment enriched with soluble factors associated with fibrosis development, such as cytokines, chemokines, and EVs production. In recent years, it has been shown that EVs participate in the initiation, and progression of the pathophysiological mechanisms associated with IPF; for example, immunomodulation, fibroproliferation, differentiation, and mesenchymal–epithelial transition through the transfer of bioactive cargo molecules, such as proteins (PD-L1, WNT5A and Fibronectin) and miRNAs (miR-328, miR-125a-5p, miR-142-3p, miR-23b-3p and miR-494-3p). Thus, EVs can modulate the functioning of the host cells, accelerating lung damage and leading to decreased gas exchange by excessive ECM deposition. Therefore, these crucial features strongly suggest that EVs play an essential role in the pathogenesis of IPF and may be useful for achieving a better understanding of the disease and identifying candidates for biomarkers for the diagnosis, prognosis of IPF. However, there are still many challenges ahead; despite the number of available studies describing how EVs and their cargo molecules play an essential role in IPF progression, the mechanism by which these EVs regulate cellular processes involved in IPF development remains to be fully elucidated. Therefore, further exploration and new studies are needed to expand the understanding of EVs and their cargo molecules in the pathogenesis of IPF. Moreover, MSC-EVs research has developed rapidly during the last decade, representing a promising therapeutic approach for IPF. These MSC-EVs represent a more successful therapeutic approach than MSCs therapy, because they present a lower risk of immunogenicity and can exert a similar therapeutic effect to MSCs by maintaining anti-inflammatory and antifibrotic properties. However, although the clinical application of MSC-EVs has been raised as a possible therapeutic option that could control the pathological state of IPF patients, there are still several limitations and several methodological issues related to the isolation, purification, preparation, and characterization of these MSC-EVs that need to be improved to allow us the clinical application of these EVs soon.

## Figures and Tables

**Table 1:** Summary of studies in animal models of IPF with therapeutic MSC-EVs.

Experimental Model	EVs Source	Cargo	Effects	Reference
Radiation-induced lung injury	hP-MSCs	miR-214-3p	Attenuates pulmonary vascular damage, inflammation, and fibrosis.	[68]
Bleomycin-induced IPF	BMSCs	miR-186	Relieves IPF by blocking fibroblasts activation by suppressing SOX4 and DKK1 expression.	[72]
*E. coli* endotoxin induced ALI	BMSCs	KGF mRNA	Restores lung protein permeability and reduces inflammation.	[71]
IRI-induced ALI	BMSCs	miR-21-5p	Decreases edema, pulmonary dysfunction, M1 polarization of alveolar macrophages, and secretion of the cytokines IL-8, IL-1β, IL-6, IL-17, and TNF-α.	[73]
Hypoxia-induced PH	BMSCs	unknown	Prevents activation of hypoxic signaling, lung inflammation, and PH development through inhibition of hypoxic STAT3 signaling.	[74]
Bleomycin-induced IPF	BMSCs	miR-29b-3p	Attenuates IPF progression by suppressing fibroblasts proliferation, migration, and differentiation through suppression of FZD6 expression.	[75]
LPS-induced ALI	BMSCs	miR-23a-3p y miR-182-5p	Attenuate lung injury, EMT, and fibrosis by inhibiting NF-κB and hedgehog pathways.	[76]
LPS-induced ALI	AD-MSCs	unknown	They reduce inflammation, alveolar septal thickening, alter macrophage phenotypes, reduce levels of the proinflammatory cytokine IL-1β, and increase anti-inflammatory IL-10.	[77]
LPS-induced ALI	AD-MSCs	miR-27a-3p	Alleviates lung injury, inhibits NF-κB activation and promotes M2 polarization of alveolar macrophages.	[78]
Bleomycin-induced IPF	hAECs	unknown	Attenuates inflammation and pulmonary fibrosis.	[79]
Bleomycin-induced IPF	MenSCs	miR-Let-7	Attenuates lung inflammation and fibrosis by regulating ROS, mtDNA damage, and NLRP3 inflammasome activation.	[80]

hP-MSCs = Placenta-derived mesenchymal stem cell; BMSCs = Bone marrow mesenchymal stem cell; AD-MSCs = adipose-derived mesenchymal stem cells; hAECs = Human amnion epithelial cells; MenSCs = Menstrual blood-derived stem cells; IPF = Idiopathic pulmonary fibrosis; ALI = Acute lung injury; LPS = Lipopolysaccharide; IRI = Ischemia/reperfusion injury; PH = Pulmonary hypertension; EMT = Epithelial–mesenchymal transition; ROS = Reactive oxygen species; DKK1 = Dickkopf-1; IL-8 = Interleukin-8; IL-1β = Interleukin-1β; IL-6 = Interleukin-6; IL-17 = Interleukin-17; TNF-α = Tumor necrosis factor alpha; IL-10 = Interleukin-10; STAT3 = signal transducer and activator of transcription 3; FZD6 = Frizzled-6; NF-κB = Nuclear factor kappa B; mtDNA= mitochondrial DNA; NLRP3= NLR family pyrin domain containing 3; SOX4= SRY-box transcription factor 4.

**Table 2:** Summary of studies in vitro models of IPF and other lung diseases with therapeutic MSC-EVs.

EVs Source	Cargo	Target	Effect	Reference
hAECs	unknown	HLF activated with TGF-β	Inhibit fibroblasts activation	[79]
BMSCs	miR-186	Fibroblasts LL29	Inhibit fibroblasts activation by supression of SOX4 and DKK1	[71]
BMSCs	interaction of Thy-1 with beta integrins	CCL-210 (HLF)	Blocks myofbroblastic diferentiation	[81]
BMSCs	miR-29b-3p	LL29	Inhibit the activation of fibroblasts through FZD6	[75]
BMSCs	miR-21-5p	Dendritic cells activated with LPS	Increased production of antiinflammatory cytokines Reduced synthesis of proinflammatory cytokines, such as IL-6 and IL-12 Decreased migratory capacity	[82]
BMSCs	miR-182-5p and miR-23a-3p,	MLE-12 cell activated with LPS	Inactivate NF-κB and hedgehog pathways Reverts EMT	[76]
Human lung MSCs	unknown	IB3-1 cells activated with TNF-α	Attenuate the expresión of inflammatory cytokines Increase antioxidant intrinsic defenses	[83]
MenSCs	miR-Let-7	MLE-12 cells activated with BLM	Inhibits pulmonary fibrosis through regulation of ROS, mtDNA damage and NLRP3 inflammasome activation.	[80]
AD-MSCs	unknown	Macrophage activated with LPS	Suppressed the activation of proinflammatory genes IL-6, IL-1β, TNF-α	[77]
AD-MSCs	miR-27a-3p	Macrophage activated with LPS	Inhibits NF-κB activation and promotes M2 polarization.	[78]

BMSCs = Bone marrow mesenchymal stem cell; AD-MSCs = adipose-derived mesenchymal stem cells; hAECs = Human amnion epithelial cells; MenSCs = Menstrual blood-derived stem cells; LPS = Lipopolysaccharide; HLF = Human Lung Fibroblasts; EMT = Epithelial–mesenchymal transition; ROS = Reactive oxygen species; DKK1 = Dickkopf-1; IL-1β = Interleukin-1β; IL-6 = Interleukin-6; IL-12 = Interleukin-12; TNF-α = Tumor necrosis factor alpha; FZD6 = Frizzled-6; NF-κB = Nuclear factor kappa B; SOX4= SRY-box transcription factor 4; MLE-12 = murine lung epithelial cell; mtDNA= mitochondrial DNA; NLRP3= NLR family pyrin domain containing 3.

## Data Availability

Not applicable.
